# Morphometrical Study of the European Shorthair Cat Skull Using Computed Tomography

**DOI:** 10.3390/vetsci8080161

**Published:** 2021-08-10

**Authors:** Joana Ramos, Inês Viegas, Hugo Pereira, João Filipe Requicha

**Affiliations:** 1 Faculty of Veterinary Medicine, Lusófona University, Av. do Campo Grande 376, 1749-024 Lisbon, Portugal; joanaiar@hotmail.com (J.R.); inesviegas@gmail.com (I.V.); hugobiwan@yahoo.es (H.P.); 2 Animal and Veterinary Research Center and AL4AnimalS, Department of Veterinary Sciences, University of Trás-os-Montes e Alto Douro, Quinta de Prados, 5000-801 Vila Real, Portugal

**Keywords:** European shorthair, skull, cranium, computed tomography, morphometric analysis

## Abstract

This study aimed to perform a morphometric analysis of the skull of the European shorthair cat by using computed tomographic images. Thirty-seven computed tomography (CT) studies of healthy cats’ heads were used for linear measurements and index calculations of the skull and cranium. The following values were determined: skull length = 8.94 ± 0.45 cm, cranial length = 8.21 ± 0.42 cm, nasal length = 0.73 ± 0.17 cm, cranial width = 4.28 ± 0.26 cm, cranial index = 52.18 ± 3.75%, internal height of cranium = 2.88 ± 0.29 cm, external height of cranium = 3.35 ± 0.12 cm, internal length of the cranium = 5.53 ± 0.28 cm, external length of the cranium = 6.32 ± 0.28 cm, internal cranium index = 45.62 ± 4.77%, external cranium index = 53.06 ± 2.07%, internal cranium and skull index = 61.93 ± 2.38%, external cranium and skull index = 70.70 ± 1.72%, width of the foramen magnum = 1.34 ± 0.07 cm, height of the foramen magnum = 1.01 ± 0.09 cm, and foramen magnum index = 75.37 ± 5.76%. It was also found that the population was homogeneous, with the exception of nasal length (NL), and that there was a sexual dimorphism present, with males exhibiting higher dimensions. This work contributed to characterizing the morphometry of the cranium and skull of the domestic cat, a knowledge of utmost importance for the diagnosis and treatment of conditions affecting this complex anatomical region.

## 1. Introduction

The skull is divided into the cranium and face, including the mandible and the hyoid bone [1,2]. Bones of the cranium comprise the occipital, presphenoid, basisphenoid, pterygoid, ethmoid, vomer, temporal, parietal and frontal bones [2,3,4,5,6]. The conformation of the domestic cat’s head (*Felis catus*, Linnaeus 1758) depends on the shape of the skull and is strongly related to the specific skeletal properties of the breed [7,8]. In most cats, the face is relatively small; however, in certain Eastern breeds, with special focus on the Siamese breed, the skull is elongated with a triangular shape (dolichocephalous), in contrast to Persian cats, which are brachycephalic [1,7,9].

Phenotypically, cats have a globular jaw and a rounded skull, surrounded by a small sagittal crest, corresponding very closely to the contours of the cranium, convex and protruding zygomatic arches, and a relatively short face, corresponding to approximately 20% of total head length [1].

Currently, radiographic evaluation of the skull has been replaced by advanced imaging techniques, such as computed tomography (CT) and magnetic resonance imaging. These techniques allow a rigorous evaluation of the skull’s complex anatomy with reduced exposure to radiation [10,11,12], paving the way for novel morphometric studies [13,14,15].

There are several diseases directly related to the conformation of the skull in felines, such as external hydrocephalus [16,17], meningocele and meningoencephalocele [18,19,20], congenital fusion of the hard palate with an extension of the presphenoid bone which culminates in bilateral osseous atresia of the choanae [21], and facial, dental, and neurocranial abnormalities associated with brachycephaly [22], traumatic injuries or bone neoplasia [23].

The objective of this work was to characterize the morphology of the cranium and skull of the European shorthair cat breed and to validate the use of computed tomographic images for this purpose.

## 2. Materials and Methods

### 2.1. Study Population

The study population comprised a convenience sample of 37 European shorthair cats, ranging from 1 to 17 years (average of 8.3 years, median of 8 years), 19 females and 18 males, which underwent CT examination of the head at Hospital Veterinário do Restelo (Lisbon, Portugal). The selected CT images were retrospectively evaluated, and no animal was used or handled for the purpose of this study.

CT selection followed the criteria: (i) a minimum age of 12 months, assuming skeletal development at this age, (ii) the cats did not present any traumatic or gross pathological structural osseous changes that could interfere with the identification of the anatomical landmarks, and (iii) the images had suitable quality and definition of the bony contours of the head in the three main reconstructions (transverse, dorsal and sagittal).

### 2.2. Computed Tomography of the Head

CT images of the studied heads were obtained by using a single-slice helical device HiSpeed LX/i (General Electric Company, Medical Systems, Boston, MA, USA). Images were acquired in a bone algorithm (window level between 200 and 2250 Hounsfield units (HU) and bone filter to reduce the noise).

Animals were placed in ventral recumbency, and their correct positioning was evaluated by performing topograms and corrected when necessary. They were induced with propofol (Propofol Lipuro 10 mg/mL, B.Braun Portugal, Barcarena, Portugal) at a dose of 3–4 mg/kg intravenously, and the maintenance of anesthesia was carried out with volatile isoflurane mixed with oxygen.

### 2.3. Measurements of the Skull and Cranium

**Figure 1:**
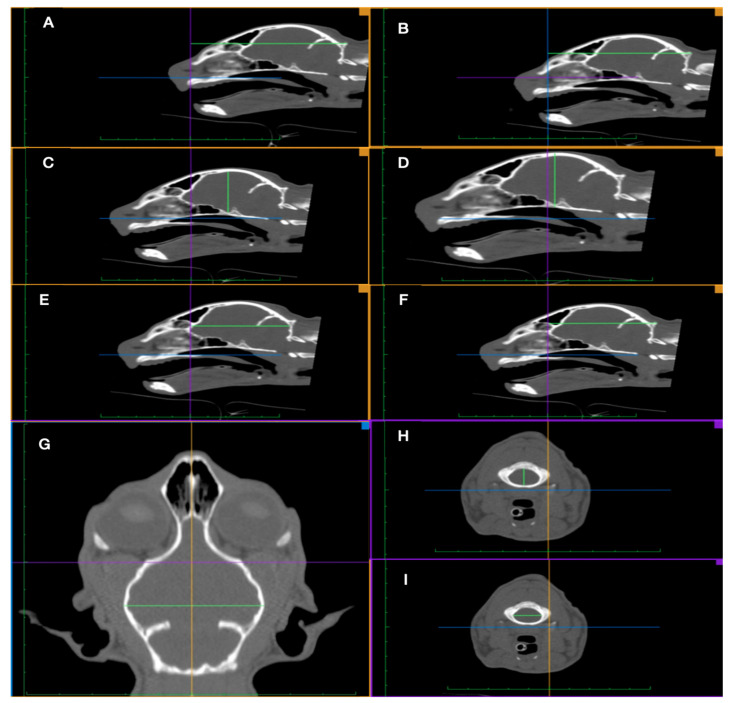
Schematic representation of the measurements and anatomical landmarks used for the morphometric study of the skull and cranium. (**A**)—Skull length (SL), in the sagittal plane, from the rostral border of the nasal bone to the external occipital protuberance (this is subdivided between cranial and nasal length). (**B**)—Cranial length (CL), in the sagittal plane, from the external occipital protuberance to the caudal limit of the nasal bone. (**C**)—Internal height of the cranium (IHC), in the sagittal plane, from the deepest indentation of the sella turcica directly dorsal to the inner layer of the base of the cranium to the most dorsal surface of the cranium. (**D**)—External height of the cranium (EHC), in the sagittal plane, being identical to the IHC but in the external face of the bone surfaces in question. (**E**)—Internal length of the cranium (ILC), in the sagittal plane, from the deepest indentation of the fronto-ethmoidal junction to the middle of the distal surface of the cranium at the level of the cerebral surface of the external occipital protuberance. (**F**)—External length of the cranium (ELC), in the sagittal plane, being identical to the ILC but of the external surface of the bones in question. (**G**)—Cranial width (CW), in the dorsal plane, between the two most lateral points of the cranium. (**H**)—Width of the foramen magnum (FMW), in the transverse plane, by the identification of the two parallel points more lateral of the foramen magnum. (**I**)—Height of the foramen magnum (FMH), in transverse plane, with the vertical height being obtained in the center of the foramen magnum.

The anatomical landmarks and the linear measurements, demonstrated in Figure 1, were selected according to Monfared (2013) and Uddin and colleagues (2013) [8,24]. The linear measurements and indexes were the skull length (SL), cranial length (CL), nasal length (NL), cranial width (CW), cranial index (Ci), internal height of cranium (IHC); external height of cranium (EHC), internal length of the cranium (ILC), external length of the cranium (ELC), internal cranium index (ICi), external cranium index (ECi), internal cranium/skull index (ICSi) external cranium/skull index (ECSi), foramen magnum width (FMW), foramen magnum height (FMH) and foramen magnum index (FMi).

CT images were processed in the format standardized by the Digital Image Communication in Medicine (DICOM) system. Linear measurements were performed using the DICOM imaging software Osirix Lite (Pixmeo, Bernex, Switzerland) using a bone window with a range between 200 and 1000 HU to optimize the contrast. This software allows, simultaneously, the evaluation in three anatomical planes, namely the transverse, sagittal and dorsal plane, which allows the delimitation of anatomical structures with greater precision.

In order to reduce the analysis margin of error, three measurements of each studied parameter were performed. The measurements were performed by the same operator (to reduce interpersonal errors), and each measurement of each parameter was performed at different times, in order to reduce intrapersonal errors. Then, the arithmetic average of the measurements was calculated.

In order to perform the linear measurements (Figure 1), the images were centered and aligned based on the following anatomical reference points: the temporomandibular joint and/or tympanic bulla in the transverse plane, the hard palate in the median sagittal plane and the nasal septum in the dorsal plane. In the case of the FMW and FMH measurements obtained in the transverse plane, the median sagittal plane of the image was aligned by the foramen magnum. The nasal length (NL) was obtained through the formula “NL = SL − CL”. In addition to the performed linear measurements, the indices relating them were determined: cranial index (Ci) = CW/CL × 100; internal cranium index (ICi) = IHC/ILC × 100; external cranium index (ECi) = EHC/ELC × 100; internal cranium and skull index (ICSi) = ILC/SL × 100; external cranium and skull index (ECSi) = ELC/SL × 100 and the foramen magnum index (FMi) = FMH/FMW × 100.

### 2.4. Statistical Analysis

Statistical analysis was performed using the software SPSS Statistics, version 22.0 (IBM Corp., Armonk, NY, USA). Normality was verified through Kolmogorov–Smirnov tests, which were non-significant for all the tested variables [25]. Descriptive statistics included mean, mean standard deviation, variance and coefficient of variation, with a confidence interval (CI) of 95%, and were computed for the overall population, and for both genders separately.

The inferential statistical analysis began by ruling out significant differences between the three measurements performed for each variable (linear measurement), for which we used one-way ANOVA tests (non-significant for all the computed variables).

Finally, independent sample *t*-tests with a significance level of 95% were computed to assess differences between genders for all the relevant variables.

## 3. Results

### 3.1. Measurements of the Skull and Cranium

By using the one-way ANOVA statistical method, it was observed that there was no statistically significant difference between the three measurements performed in each animal and per parameter.

Considering the sample simple size, a standard normal distribution was assumed [25]. The descriptive statistical analysis for this sample of European shorthair cats is presented in Table 1. To evaluate the homogeneity of the study population, the coefficient of variation was calculated.

The parameters with the lowest coefficient of variation were ECSi (2.436), EHC (3.494), ICSi (3.836), ECi (3.907), ELC (4.447), ILC (4.987), SL (5.077), CL (5.079), FMW (5.385), CW (5.988), Ci (7.194), FMi (7.646), FMH (9.028), IHC (9.937) and ICi (10.461). The NL, with the highest coefficient of variation, stands out with a value of 22.814.

### 3.2. Analysis of the Skull and Cranium Measurements Relating to Gender

The results of the descriptive statistical study related to gender are shown in Table 2. In order to evaluate the two independent samples, male and female populations, the t-test was performed (Table 3). The coefficient of variation was calculated again to evaluate the homogeneity of the study population by gender.

Performing the coefficient of variation, the following results were obtained: ICC of 1.976 in females, ECSi of 2.342 in males, SL of 2.397 in females, ELC of 2.426 in females, EHC of 2.817 in females, ICSi of 3.139 in males, ECi of 3.147 in females, CL of 3.156 in females, CW of 3.421 in females, SL of 3.576 in males, EHC of 3.589 in males, ILC of 3.634 in females, CL of 3.922 in males, ICSi of 3.960 in males, ILC of 4.840 in males, ICi of 5.391 in males, FMW of 6.268 in males, FMi of 6.932 in males, CW of 7.962 in males, Ci of 8.217 in males, FMi of 8.401 in females, FMH of 8.670 in males, FMH of 9.491 in females, Ci of 10.162 in females, ICi of 11.831 in females and IHC of 12.948 in females. Again, NL presents higher values of the coefficient of variation, it being 19.322 in males and 23.547 in females.

## 4. Discussion

This work contributed to the knowledge about the morphology of the skull and cranium of healthy European shorthair cats. Considering the large age range from 1 to 17 years, the obtained results do not allow us to make inferences about the evolution of the studied parameters at each specific age, but only to describe the morphological pattern of adult cats.

The internal cranium index (ICi) was 7.438% lower than the external cranium index (ECi), corresponding with the thickness of cranial bones. The length of the cranium was more than half the length of the skull. The index of the foramen magnum (FMi) was high, demonstrating the similarity of the values between the height of the foramen magnum (FMH) and the foramen magnum width (FMW), showing the elliptic but almost round shape of this foramen.

Evaluation of the coefficient of variation of the skull and cranium parameters between individuals allowed us to establish that the population is homogeneous; for example, low coefficients of variation for ECSi and ICi, between 2.436% and 10.461%, respectively. Nasal length was an exception, having a high coefficient of variation (22.814%), demonstrating the heterogeneity of the studied population regarding nasal length.

When assessing the homogeneity of the population according to gender, the obtained results were identical to those of the total population, with low coefficient of variation values (between 1.976% and 12.948%, corresponding to ECSi in females and IHC in females, respectively). The exception was again the nasal length (NL) (19.322% in males and 23.547% in females), revealing that females presented a greater variability in relation to males.

The comparison between genders showed that there is a statistically significant mean value variation between males and females (Table 3). In SL, CL, NL, EHC, ILC and ELC, means were higher in males, contrary to Ci, ICi, ECi, ICSi and ECSi, which were higher in females. Thus, males have a longer skull and cranium compared to females. These results are in agreement with the expected sexual dimorphism observed in felines [26,27], including in domestic cats [9]. An interesting fact is the presence of high sexual dimorphism in linear measurements of skull length (SL), cranial length (CL) and external length of the cranium (ELC), in which the results differ significantly (*p* < 0.001) between genders. Some of those features are possibly influenced by sex hormones [28]; unfortunately, this issue could not be evaluated in this study, due to the lack of information about neutering. Further studies comparing the effect of this procedure with morphometric parameters should be performed.

The present morphometric study based on CT images of 37 cats revealed lower mean values than those described in a study in which measurements were made directly on bone surfaces after maceration of the head [9]. This discrepancy of data could be justified by the different geographical location of these populations and the distinct genetic background, which is also known to affect the head morphometry [29,30]. In addition, the technique used in this study allows rigorous measurements with three decimal places, as well as a correct observation of bone features in order to properly establish the anatomical landmarks. Another advantage of computed tomography is the visualization of intracranial planes, which is not possible in postmortem specimens without partially damaging the osseous anatomy.

Regarding the parameters of the skull, in comparison to those obtained by Monfared (2013), who performed a morphometric study on the heads of Persian cats, significant discrepancies were observed in skull length (SL), nasal length (NL) and cranial length (CL) [24]. The obtained SL and NL were lower, in contrast to CL, which was higher than in the Persian cats. In addition, the cranial width (CW) was quite similar among both studies, even though Monfared (2013) demonstrated that the Persian breed has a very characteristic anatomical conformation of the head [24]. The fact that Persians are brachycephalic could justify the smaller cranial length (CL), in contrast to the cranial width (CW), which was similar to the studied European shorthair specimens. The fact that the cranial width (CW) presents very similar values is interesting and may suggest that results associated with skull width are independent of head conformation.

Further studies could be performed to evaluate the influence of aging on the anatomical dimensions and proportions of these anatomical regions. It is known that in the domestic cat, the fusion of the ossification centers occurs between 14 and 20 months and can extend beyond 20 months [27]; however, it is often observed that cranial suture ossification does not occur, even in geriatric cats [1]. Additionally, in a study that evaluated biometric characteristics in juvenile, subadult and adult domestic cats, it was observed that the skull changes dynamically with age. It was shown that the ratio of the total cranium breadth to the total cranium length does not change in the three age stages of the individuals. However, the ratio of the cranial base length to that of the cranium increases. The cranium itself starts to broaden out in the time period between the subadult and adult age stages in relation to its height [31]. In the present work, this evaluation was not possible, because of the insufficient sample size and variability regarding head size.

This preliminary and exploratory study allowed us to demonstrate the feasibility and reliability of using digital 3D multiplanar reconstruction planes in examining the morphometry of the skull. This technique is practical, simple and low-cost compared to other methods using osteological collection which implies performing anatomical dissections. In the future, it will be pertinent to extend the study population to include individuals of other breeds, namely brachycephalic and dolichocephalic breeds, and evaluate any significant differences in the morphometric parameters of the skull.

## 5. Conclusions

This study showed that the evaluated morphometric parameters were homogeneous, with the exception of nasal length, and a sexual dimorphism was found, this being that the males exhibiting higher dimensions. This work contributed to characterizing the morphology of the skull of the domestic cat, which is of utmost importance for the diagnosis and treatment of conditions affecting this complex anatomical region.

## Figures and Tables

**Table 1:** Results of the descriptive statistics analysis of the morphometric parameters of the skull and cranium obtained in the population of European shorthair cats.

			CI of 95%		Median (cm)	Highest Value (cm)	Lowest Value (cm)	Standard Deviation	Coefficient of Variation
		Mean (cm)	Higher Value (cm)	Lower Value (cm)					
Skull Parameters	SL	8.942	9.094	8.791	8.867	9.821	8.283	0.454	5.077
	CL	8.210	8.349	8.071	8.090	9.047	7.529	0.417	5.079
	NL	0.732	0.788	0.677	0.741	1.018	0.320	0.167	22.814
	CW	4.275	4.361	4.190	4.282	4.681	3.112	0.256	5.988
	Ci	52.182	53.433	50.930	52.923	58.198	36.680	3.754	7.194
Cranium Parameters	IHC	2.878	2.973	2.783	2.851	4.466	2.479	0.286	9.937
	EHC	3.349	3.388	3.310	3.328	3.670	3.140	0.117	3.494
	ILC	5.534	5.626	5.442	5.527	6.041	5.071	0.276	4.987
	ELC	6.319	6.413	6.225	6.283	6.896	5.835	0.281	4.447
	ICi	45.617	47.209	44.026	45.525	70.167	37.964	4.772	10.461
	ECi	53.055	53.746	52.364	53.293	57.925	47.939	2.073	3.907
	ICSi	61.932	62.725	61.140	62.487	67.810	57.833	2.376	3.836
	ECSi	70.703	71.277	70.128	70.875	73.747	67.261	1.722	2.436
	FMW	1.337	1.361	1.313	1.361	1.501	1.191	0.072	5.385
	FMH	1.008	1.038	0.978	1.019	1.163	0.753	0.091	9.028
	FMi	75.373	77.295	73.452	75.733	85.828	60.407	5.763	7.646

**Table 2:** Results of the descriptive statistics analysis of the morphometric parameters of the skull and cranium obtained in the population of European shorthair cats relating to gender.

		Gender	CI of 95%			Median (cm)	Highest Value (cm)	Lowest Value (cm)	Coefficient of Variation
			Mean (cm)	Higher Value (cm)	Lower Value (cm)				
Skull Parameters	SL	M	9.312	9.477	9.146	9.384	9.821	8.670	3.576
		F	8.593	8.692	8.494	8.581	8.900	8.283	2.397
	CL	M	8.515	8.681	8.349	8.508	9.047	7.835	3.922
		F	7.922	8.042	7.801	7.963	8.445	7.529	3.156
	NL	M	0.797	0.873	0.721	0.805	1.018	0.538	19.322
		F	0.671	0.747	0.595	0.713	0.904	0.320	23.547
	CW	M	4.283	4.453	4.114	4.352	4.681	3.112	7.962
		F	4.268	4.338	4.197	4.277	4.521	4.028	3.421
	Ci	M	50.348	52.406	48.291	50.137	55.588	36.680	8.217
		F	53.918	55.046	52.790	53,485	58.198	50.485	10.162
Cranium Parameters	IHC	M	2.827	2.889	2764	2.809	3.018	2.479	4.457
		F	2.927	3.109	2.744	2.866	4.466	2.665	12.948
	EHC	M	3.399	3.459	3.338	3.370	3.670	3.264	3.589
		F	3.301	3.346	3.256	3.297	3.474	3.140	2.817
	ILC	M	5.682	5.818	5.545	5.728	6.041	5.204	4.840
		F	5.394	5.489	5.300	5.409	5.694	5.071	3.634
	ELC	M	6.504	6.638	6.370	6.535	6.896	6.030	4.136
		F	6.143	6.215	6.071	6.154	6.364	5.835	2.426
	ICi	M	43.513	44.680	42.347	44.113	46.597	37.964	5.391
		F	47.611	50.326	44.896	46.588	70.167	43.285	11.831
	ECi	M	52.314	53.419	51.209	52.314	56.062	47.939	4.249
		F	53.757	54.572	52.942	53.506	57.925	50.272	3.147
	ICSi	M	61.014	61.966	60.061	61.563	63.526	57.833	3.139
		F	62.803	64.001	61.604	63.321	67.810	58.779	3.960
	ECSi	M	69.852	70.665	69.039	69.811	73.178	67.261	2.342
		F	71.508	72.189	70.828	71.615	73.747	69.017	1.976
	FMW	M	1.356	1.398	1.313	1.361	1.501	1.215	6.268
		F	1.320	1.346	1.294	1.318	1.421	1.191	4.167
	FMH	M	1.015	1.059	0.972	1.019	1.163	0.753	8.670
		F	1.001	1.047	0.955	1.028	1.118	0.827	9.491
	FMi	M	74.924	77.507	72.341	75.436	81.484	60.407	6.932
		F	75.798	78.867	72.729	76.055	85.828	62.740	8.401

**Table 3:** Results of the t-test for independent samples in which the significance is less than 0.05, with a statistically significant difference in measurements between the males and females.

		Significance	Difference (Female-Male)	CI 95% Mean (Difference Female-Male)	
				Lower Value	Highest Value
Skull Parameters	SL	0.000	−0.719237	−0.906791	−0.531683
	CL	0.000	−0.593232	−0.789278	−0.397186
	NL	0.019	−0.126005	−0.230235	−0.021774
	Ci	0.003	3.569591	1.341895	5.797286
Cranium Parameters	EHC	0.009	−0.097626	−0.169721	−0.02553
	ILC	0.001	−0.28746	−0.446053	−0.128867
	ELC	0.000	−0.360853	−0.505145	−0.216561
	ICi	0.007	4.097600	1.187598	7.007603
	ECi	0.032	1.443121	0.129283	2.75696
	ICSi	0.020	1.788865	0.301497	3.276233
	ECSi	0.002	1.656587	0.638324	2.67485

## Data Availability

Not applicable.
